# Buffalo City Dispensary Appointments for 1863

**Published:** 1863-01

**Authors:** 


Buffalo City Dispensary Appointments for 1863.—*Physicians:* 1st Ward, Dr. E. E. Brownell; 2d Ward, Dr. P. H. Strong; 3d Ward, Dr. J. Boardman; 4th Ward, Dr. J. R. Lothrop; 5th Ward, Dr. J. Hauenstein; 6th Ward, Dr. James B. Samo; 8th Ward, Dr. Sandford Eastman; 9th Ward, Dr. C. C. Wyckoff; 10th Ward, Dr. J. Whittaker; 11th Ward, Dr. L. P. Dayton; 12th Ward, Dr. Henry Nichell; 13th Ward, Dr. Wm. Ring.

*Consulting Physicians.*—Drs. Thomas F. Rochester and George N. Burwell.

*Consulting Surgeons.*—Drs. James P. White and Julius F. Miner.

*Apothecary.*—William H. Peabody, Clarendon Hotel Block, corner Main and South Division streets.

---

☞ By reference to our advertising columns, it will be seen that D. Clinton Hicks, Esq., announces an important reduction in the price of tuition at his well-established and prosperous Institution, No. 104 Main street, opposite the Metropolitan Theatre. Book keeping complete, in all the different departments of commerce, is now taught by him for twenty-five dollars only. Arrangements have also been consummated for the delivery of a course of Lectures, and it is his determination to render his College worthy in an eminent degree, of the support of the public. Prof. Hicks is a gentleman of fine talents, unceasing industry, and commendable enterprise, and we cordially wish him success.

